# Untargeted Global Metabolomic Analysis Reveals the Mechanism of Tripropylamine-Enhanced Lycopene Accumulation in *Blakeslea trispora*

**DOI:** 10.3389/fbioe.2021.673225

**Published:** 2021-06-02

**Authors:** Yanlong Wang, Yulong Wang, Yicun Wang, Xin Chen, Cunping Liu, Meng Zhang, Keying Liu, Yuechao Zhao, Zexu Li

**Affiliations:** ^1^Collaborative Innovation Center for Birth Defect Research and Transformation of Shandong Province, Jining Medical University, Jining, China; ^2^College of Teacher Education, Qilu Normal University, Jinan, China; ^3^Shandong Institute for Product Quality Inspection, Jinan, China

**Keywords:** *Blakeslea trispora*, tripropylamine, untargeted metabolome, quorum sensing, lycopene

## Abstract

We previously determined that the cyclase inhibitor tripropylamine (TPA) significantly enhances lycopene accumulation in *Blakeslea trispora*. To elucidate the mechanism of TPA-enhanced lycopene accumulation, the untargeted metabolome of *B. trispora* treated with TPA was analyzed by UHPLC-Q-TOF/MS. Forty-two differential metabolites were identified, of which 15 significantly differential metabolites meeting the following parameters were screened: variable importance for the projection > 1, *P* < 0.05, and fold change > 1.5. The down-regulated metabolites were mainly cyclic dipeptides, bacteriostatic compounds, and lipids, while the up-regulated metabolites were mainly unsaturated fatty acid. Furthermore, the bacteriostatic ability was poor, the extracellular and intracellular pH levels were high, and hyphae with vesicles were swollen locally in *B. trispora* after treatment with TPA. Our data suggest that the TPA enhances lycopene accumulation not only by inhibiting the cyclization of β-carotene but also by down-regulating cyclic dipeptides for quorum sensing; up-regulating unsaturated fatty acids, 1-palmitoyl-2-hydroxy-sn-glycero-3-phosphoethanolamine, and 4-hydroxybenzoate and down-regulating choline, resulting in locally swelling mycelium with vacuoles; and down-regulating bacteriostatic metabolites for metabolic flux redistribution.

## Introduction

Lycopene, a well-known member of the carotenoids, exhibits strong antioxidant activity. It has been extensively used to prevent and treat prostate, breast, pancreatic, and other cancers, as well as cardiovascular diseases (Rodriquez-Concepcion et al., 2018; Caseiro et al., 2020). *Blakeslea trispora*, a filiform aerobic fungus (class Zygomycetes, order Mucorales, family Choanophoraceae), has been proposed as an ideal natural source of lycopene. During its fermentation process, the “plus” and “minus” type strains mate to generate trisporic acid from the “plus” type and stimulate lycopene synthesis by the “minus” type (Wang et al., 2016a).

During the carotenoid biosynthesis process in *B. trispora*, phytoene is catalyzed by phytoene desaturase to generate lycopene. Eventually, the lycopene is cyclized into γ-carotene and β-carotene by lycopene cyclase. Thus, lycopene is an intermediate of the carotenoid biosynthetic pathway. To enhance lycopene accumulation, lycopene cyclase should be suppressed. Two categories of chemicals, nitrogen-containing heterocyclic compounds and amines, have been reported as cyclase inhibitors that efficiently block the cyclization process. 2-Methyl imidazole and 2-isopropylimidazole are both effective in inhibiting lycopene cyclase in *B. trispora* (Pegklidou et al., 2008; Wang et al., 2014). However, amines also act as inducers for the enzyme(s) in the carotenoid biosynthesis pathway (Wang et al., 2016a). Thus, amines could significantly enhance lycopene accumulation in *B. trispora*. For example, 2-(4-chlorophenylthio) triethylamine hydrochloride increases the lycopene content from 24.87 to 412.41 mg/gDW (Hsu et al., 1974). Comparative evaluation of imidazole, pyridine, piperidine, triethylamine, and nicotinic acid has shown that triethylamine increases lycopene yield from 34.74 to 269.66 mg/L in *B. trispora*, which is second only to piperidine (Choudhari et al., 2008).

Our previous work found that tripropylamine (TPA) is an effective lycopene-enhancing compound, especially when the concentration is 1.8 g/L. It could increase the lycopene and total carotenoid contents to 83.2 and 92.4 mg/gDW and 315.8- and 5.9-fold increases, respectively, compared with the contents in untreated controls (Wang et al., 2016a). Gene expression analysis showed that all the tested genes, especially genes encoding 3-hydroxy-3-methylglutaryl coenzyme A reductase and isopentenyl pyrophosphate isomerase in the mevalonate (MVA) pathway, were up-regulated. Additionally, we previously genetically manipulated the bifunctional protein gene *carRA*, and *carR*- and *carRA*-knockout and *carA*-overexpressing strains were obtained. However, the total carotenoid content decreased in these strains because of negative regulation via the MVA pathway, while TPA significantly increased the lycopene and total carotenoid content in *carR*-knockout and *carA*-overexpressing strains (Wang et al., 2017). Therefore, the addition of TPA may prove effective in increasing industrial lycopene production in *B. trispora*. However, TPA is largely toxic to humans. Thus, understanding the mechanism of action of TPA can help researchers develop a non-toxic method to enhance lycopene yield in *B. trispora*.

In this study, the mechanism of TPA-enhanced lycopene accumulation in *B. trispora* was determined using an untargeted global metabolomic analysis. We found that the TPA enhances lycopene accumulation not only by inhibiting the cyclization of β-carotene but also by down-regulating cyclic dipeptides for quorum sensing; up-regulating unsaturated fatty acids, 1-palmitoyl-2-hydroxy-sn- glycero-3-phosphoethanolamine (PPE), and 4-hydroxybenzoate and down-regulating choline, resulting in locally swelling mycelium with vacuoles; and down-regulating bacteriostatic metabolites for metabolic flux redistribution.

## Materials and Methods

### Strains and Culturing Conditions

*B. trispora* ATCC 14271(+) and 14272(-) were obtained from the American Type Culture Collection (Manassas, VA, USA) and cultivated as previously described (Wang et al., 2016a). Cells were divided into two groups, the control group and TPA-treated group, with five biological duplicates. For the control group, *B. trispora* ATCC 14271(+) and 14272(-) were fermented for 4 days, with three repeats for each sample. For the TPA-treated group, after the cultures had fermented for 2 days, TPA (1.8 g/L) was administered for 2 days (Wang et al., 2016a). The BL21 strain (*Escherichia coli* DE3) was cultivated as our previously method (Wang et al., 2016b).

### Metabolite Extraction

*B. trispora* cell cultures of control and TPA-treated groups were harvested after fermenting for 4 days and re-suspended twice using ion-free ultrapure water. Then, the cultures were dissolved in 200 μl of ion-free ultrapure water per gram of wet weight. The cultures were then crushed and homogenized by liquid nitrogen grinding and tissue grinder, respectively. Then, methanol/acetonitrile (1:1, v/v) was added, and the cells were broken up in two rounds by using a Scientz-IID sonifier (Ningbo Scientz Biotechnology Co., China) for 30 min each round. The cells were then incubated at −20°C for 1 h to precipitate protein. The samples were centrifuged at 13,000 rpm for 15 min, and the supernatants were used as the metabolite samples and stored at −80°C after freeze-drying.

### GC-MS Analysis

The metabolite samples were separated by UHPLC-Q-TOF-MS/MS using an Agilent 1290 Infinity Series UHPLC (Agilent Technologies, Santa Clara, CA, United States). UHPLC was performed at 25°C, with a linear gradient as follows: 0–1 min, 5% phase A (ddH_2_O, 25 mM ammonium acetate and 25 mM ammonia), 95% phase B (acetonitrile); 1–14 min, 35% phase A, 65% phase B; 14–18 min, 60% phase A, 40% phase B; and 18–23 min, 5% phase A, 95% phase B, with a flow rate of 0.3 ml/min. Time-of-flight mass spectrometry was performed using a hybrid triple quadrupole (Triple TOF 5600 system, AB SCIEX, Framingham, United States) with electrospray ionization operated in both positive and negative modes. The TOF MS scanning *m/z* range was 60–1,000 Da, and the product ion scanning *m/z* range was 25–1,000 Da. The MS spectrum was obtained using information-dependent acquisition with high sensitivity.

### Bioinformatics Analysis

The raw MS data were converted to mzXML files using ProteoWizard MSConvert and processed using XCMS software (Waters) for feature detection, retention time correction, and alignment (Benton et al., 2015). The metabolites were identified by accurate masses (<25 ppm) and matched with the standards database built by Shanghai Applied Protein Technology (Shanghai, China). After pareto-scaling, multidimensional statistical analyses [including principal component analysis (PCA), partial least squares discriminant analysis (PLS-DA), and orthogonal partial least squares discriminant analysis (OPLS-DA)] were implemented in SIMCA-P 14.1 (Umetrics, Umea, Sweden). Univariate statistical analyses [including Student’s *t*-test and fold change (FC) analysis] were implemented in R programming language. For multidimensional statistical analyses, variable importance for the projection (VIP) was used to measure the degree of influence of each metabolite. The values VIP > 1 and *P* < 0.1 were set to determine differential metabolites, and VIP > 1, *P* < 0.05, and FC > 1.5 were set to determine significantly differential metabolites.

Hierarchical clustering and correlation analysis of differential metabolites were performed to accurately screen for ionic metabolites and measure the correlation of significantly differential metabolites. Kyoto Encyclopedia of Genes and Genomes (KEGG) pathway enrichment was performed using the KEGG database^1^.

### pH, Mycelial Morphology, and Bacteriostatic Ability Analysis

*B. trispora* ATCC 14271(+) and 14272(-) were fermented for 2 days, and TPA (1.8 g/L) was added to the cultures. After the samples were shaken at 28°C for 0, 1, 4, 8, 16, 32, and 64 h, the cells were harvested and lysed by using Scientz-IID sonifier. Then, the pH of the cells and fermentation was measured with a pH meter. To detect the vesicles structure in cells, hyphae were stained with 10 μM Mito-tracker Green (MG) (maximum excitation wavelength 490 nm; maximum emission wavelength 516 nm) (Beyotime Institute of Biotechnology, Shanghai, China) (Swift, 2003) and kept in the dark for 40 min at 33°C. Mycelial morphology was then detected with an upright fluorescence microscope (Ni-U; Nikon Co., Tokyo, Japan) after treatment with TPA for 2 days. Bacteriostatic ability were analyzed using *E. coli*; 10^7^ cfu/ml *E. coli* were added before TPA was added, and after TPA (1.8 g/L) was administered for 2 days, the cell density of *E. coli* was measured by a blood-cell-counting plate after shaking at 180 rpm, 28°C for 0, 1, 4, 8, 16, 32, and 64 h.

### Statistical Analysis

All experiments concerning data comparisons were performed three times. Statistical significance was determined using IBM SPSS Statistics 11.0 (SPSS Inc., Chicago, IL, United States), with the threshold set at *P* < 0.05.

## Results

### Quantitative Identification of Untargeted Metabolome Assay

After UHPLC-Q-TOF/MS analysis, 5,744 and 6,030 ionic peaks were extracted in positive and negative modes, respectively, and the total ion chromatograms of the control and TPA groups were obtained (Supplementary Figure 1). PCA (R2X > 0.5) and PLS-DA (Q2 > 0.5) analyses indicated that the models were stable and reliable. The OPLS-DA model of the control and TPA groups was established based on the modified PLS-DA. The score charts of PCA and OPLS-DA models (Supplementary Figure 2) showed that the TPA group evidently diverged from the control group, indicating that there were significant differences between and small variations within the two groups.

### Differential Metabolites Assay

**FIGURE 1:**
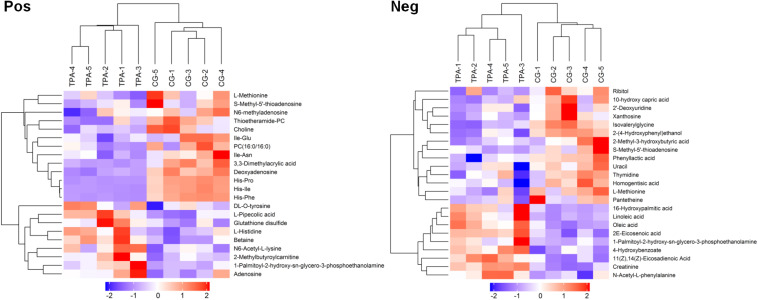
Hierarchical clustering of differential metabolites in TPA-treated *B. trispora.* Pos, positive mode; Neg, negative mode; TPA, tripropylamine treated group; CG, control group. Red and blue indicate higher and lower levels of the metabolites scaled to mean and standard deviation of raw metabolites level, respectively.

After VIP calculation, 119 and 226 differential metabolites were identified (VIP > 1 and *P* < 0.1, Supplementary Table 1) in positive and negative modes, respectively. Of these, 22 and 23 metabolites, respectively, were described (Supplementary Table 2). The hierarchical clustering of differential metabolites in positive and negative modes showed that samples of the same groups were clustered together (Figure 1). Furthermore, metabolites clustered together had similar expression patterns, suggesting that these metabolites may participate in closer reaction steps in the metabolic process.

**FIGURE 2:**
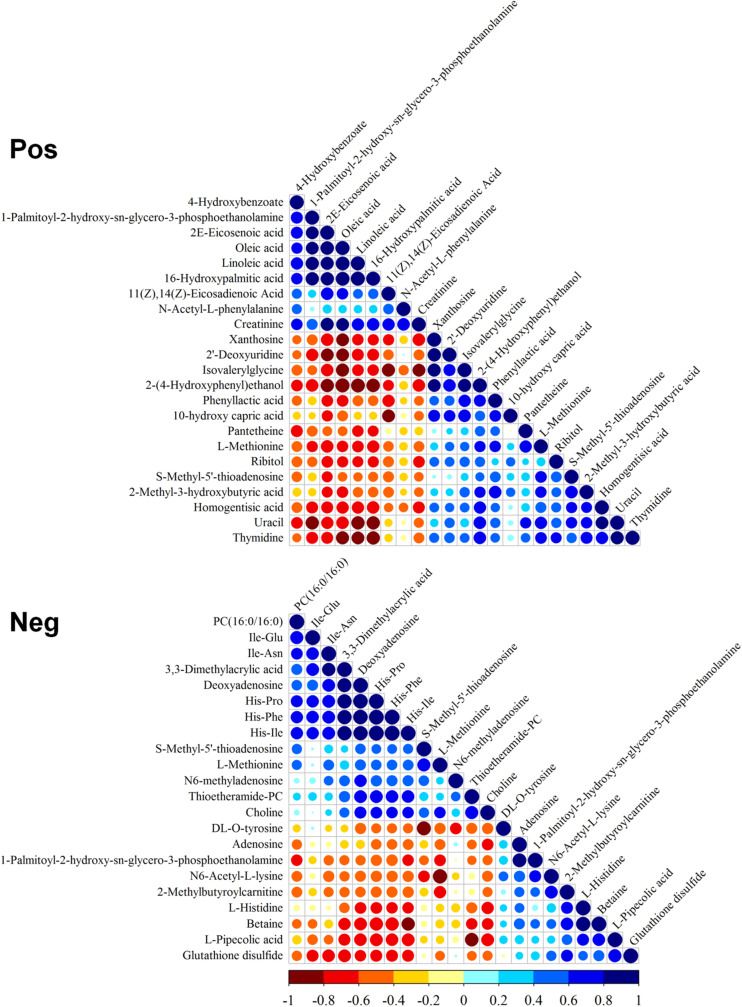
Correlation of differential metabolites. Pos, positive mode; Neg, negative mode. Blue and red indicate positive and negative correlation, respectively.

The correlation of differential metabolites in positive and negative modes was evaluated (Figure 2). The positive mode indicated that PPE was significantly positively correlated with unsaturated fatty acids [including 4-hydroxybenzoate (4-HBA), 2E-eicosenoic acid, linoleic acid, and 16-hydroxypalmitic acid] and negatively correlated with 2-(4-hydroxyphenyl)ethanol, methionine, homogentisic acid, uracil, and thymidine. The negative mode indicated that cyclic dipeptides (His-Pro, His-Ile, and His-Phe) had significant positive correlations with thioetheramide-PC (TEA-PC), PC(16:0/16:0) (lecithin), 3,3-dimethylacrylic acid, and deoxyadenosine and negative correlations with glutathione disulfide (GSSG), betaine, and PPE.

### Analysis of Significantly Differential Metabolites

To elucidate the mechanism of TPA-enhanced lycopene production in *B. trispora*, the significantly differential metabolites were screened with VIP > 1, *P* < 0.05, and FC > 1.5 as the standards, and 15 metabolites were identified (Table 1). Of these, 10 were down-regulated, and 5 were up-regulated. The down-regulated metabolites were mainly cyclic dipeptides (His-Pro, His-Ile, His-Phe, and Ile-Asn), organic acid [3,3-dimethylacrylic acid, phenyllactic acid, and isovalerylglycine (IVG)], lipids (TEA-PC and lecithin), and deoxyadenosine. The up-regulated metabolites were unsaturated fatty acids [16-hydroxypalmitic acid, linoleic acid, 2E-eicosenoic acid, and 11(Z),14(Z)-eicosadienoic acid (ω-3 PUFA)] and 4-HBA.

**TABLE 1:** Significantly differential metabolites between TPA and CG.

No.	Description	VIP	FC	*P*-value	cpdID	KEGG Map_ID
M235T253	His-Pro	20.78	0.08	0.00		
M83T260	3,3-Dimethylacrylic acid	1.14	0.14	0.00		
M252T259	Deoxyadenosine	3.27	0.18	0.00	C00559	map00230
M251T261	His-Ile	13.13	0.21	0.00		
M285T263	His-Phe	7.78	0.39	0.00		
M759T248	Thioetheramide-PC	3.15	0.48	0.01	C04873	
M165T274	Phenyllactic acid	1.50	0.55	0.02	C05607	map00360; map00960
M757T233	Lecithin	2.32	0.58	0.03	C00157	map00564; map00590; map00591; map00592; map04723
M158T336_2	Isovalerylglycine	1.88	0.62	0.00		
M228T124	Ile-Asn	2.01	0.65	0.03		
M137T64	4-Hydroxybenzoate	1.16	1.74	0.00	C00156	map00130; map00360; map00362; map00363; map00623; map00627; map00790
M307T71	11(Z),14(Z)-Eicosadienoic Acid	2.69	1.77	0.00	C16525	map01040
M309T70	2E-Eicosenoic acid	6.64	1.78	0.00		
M279T111	Linoleic acid	3.32	2.22	0.01	C01595	map00591; map01040
M271T110	16-Hydroxypalmitic acid	8.10	2.58	0.01	C18218	map00073

*Significantly differential metabolites were screened with “VIP > 1, P < 0.05, and FC > 1.5” as the standard. VIP, variable importance for the projection; FC, fold change.*

#### Cyclic Dipeptides

In this study, the significantly differential cyclic dipeptides His-Pro, His-Ile, His-Phe, and Ile-Asn were down-regulated 0. 08-, 0. 21-, 0. 39-, and 0.65-fold, respectively (Table 1). Of these, the three cyclic dipeptides containing His and His-Pro had the greatest fold change down-regulation. Furthermore, the content of histidine in the differential metabolites was up-regulated 1.24-fold (Supplementary Table 2), which may have been due to the down-regulated cyclic dipeptides.

#### Lipids and Unsaturated Fatty Acids

In this study, significantly differential lipids TEA-PC and lecithin were down-regulated 0.48- and 0.58-fold, respectively, and unsaturated fatty acids 16-hydroxypalmitic acid, linoleic acid, 2E-eicosenoic acid, and ω-3 PUFA were up-regulated 2. 58-, 2. 22-, 1. 78-, and 1.77-fold, respectively. Moreover, PPE was up-regulated 2.91-fold. The correlation analysis of differential metabolites in positive mode showed that PPE (a derivative of lysolecithin) had a significant positive correlation with these up-regulated unsaturated fatty acids (Figure 2). It further indicated that PPE may be directly produced by lysolecithin. Furthermore, choline was down-regulated in the differential metabolites, but betaine downstream was up-regulated (Supplementary Table 2).

#### Organic Acid

Among significantly differential organic acids, 4-HBA was up-regulated 1.74-fold, and others were down-regulated. Phenyllactic acid was down-regulated 0.55-fold in the phenylalanine metabolism (Table 1, map00360), indicating that, more phenylalanine than phenyllactic acid flows to produce 4-HBA. Furthermore, bacteriostatic metabolites IVG, 3,3-dimethylacrylic acid, and deoxyadenosine (Chirivi et al., 2017) were down-regulated 0. 62-, 0. 14-, and 0.18-fold, respectively.

#### Effect of TPA on Bacteriostatic Ability, pH, and Mycelial Morphology of *B. trispora*

**FIGURE 3:**
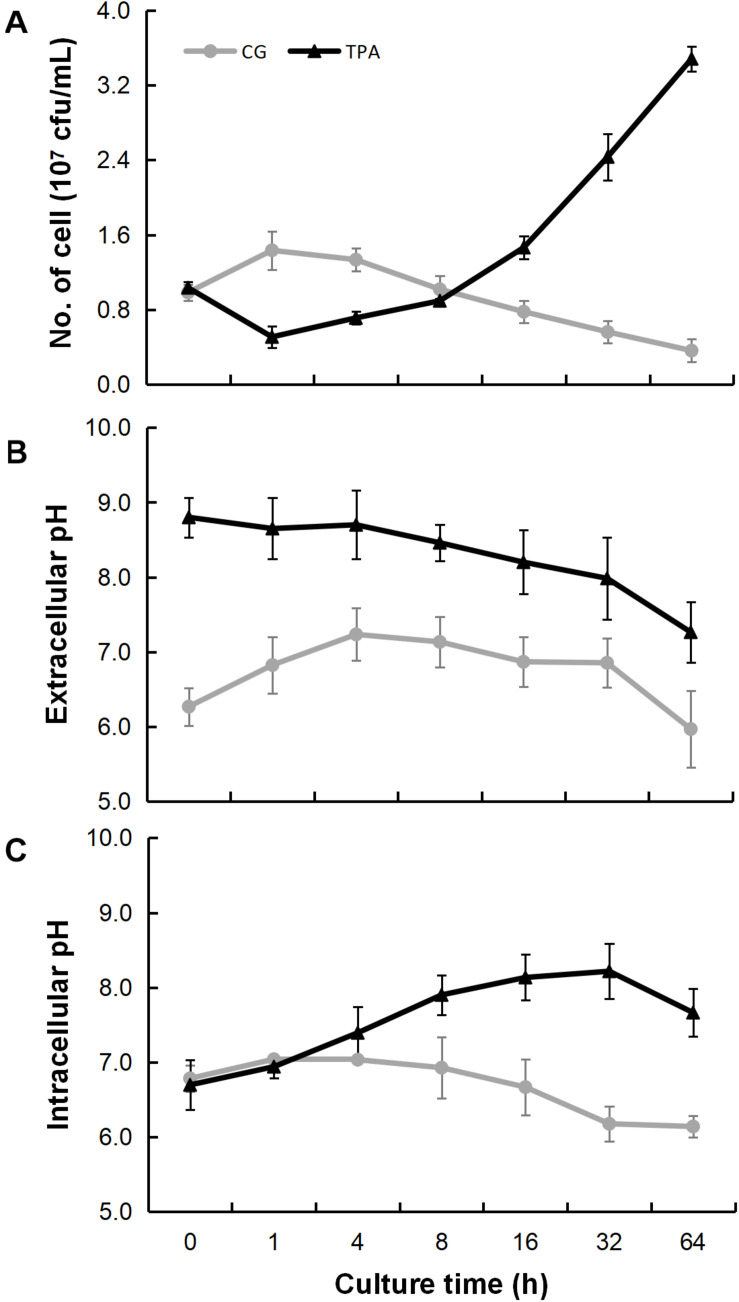
Bacteriostatic ability, extracellular pH, and intracellular pH of *B. trispora* after treatment with TPA. After the strains were fermented for 2 days, the bacteriostatic ability **(A)**, extracellular pH **(B)**, and intracellular pH **(C)** were measured after TPA treated for 0, 1, 4, 8, 16, 32, and 64 h. TPA, tripropylamine treated group; CG, control group.

**FIGURE 4:**
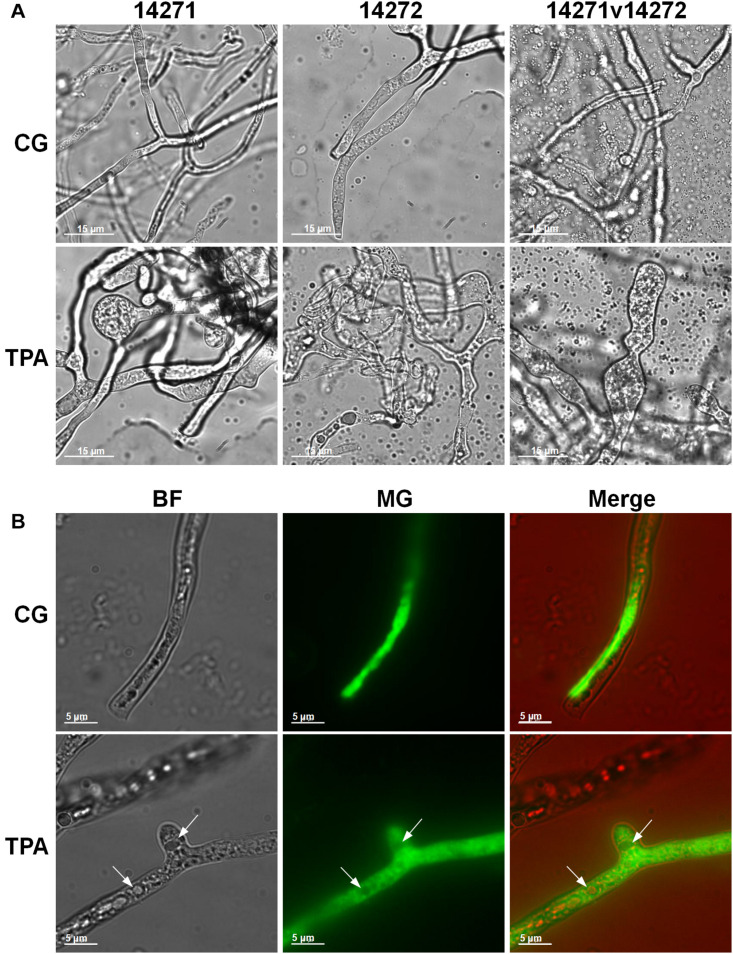
Mycelial morphology of *B. trispora* after treatment with TPA. **(A)** Mycelium in fermentation. **(B)** Fluorescence images (×200) of *B. trispora* stained with Mito-tracker Green. BF, brightfield; MG, Mito-tracker Green; TPA, tripropylamine treated group; CG, control group. For the control group, *B. trispora* ATCC 14271(+) and 14272(−) were fermented for 4 days. For the TPA-treated group, after the cultures had fermented for 2 days, TPA (1.8 g/L) was administered for 2 days.

As shown in Figure 3A, the densities of *E. coli* in the TPA group were higher than those in the control group after 8 h. However, they were lower than those in the control group in the first 8 h. The extracellular (Figure 3B) and intracellular (Figure 3C) pH levels were both higher than those in the control group. Additionally, the hyphae of 14,271, 14,272, and especially 14271v14272 were swollen (Figure 4A), and small vesicles appeared locally and were not stained with MG (Figure 4B), in contrast with mitochondria that are stained green by MG in living cells. These results indicated that these small vesicles may be independent small vacuoles that do not contain mitochondria.

## Discussion

Previously, we found that TPA could increase lycopene content by 315.8-fold in *B. trispora* (Wang et al., 2016a). However, TPA has a certain toxicity, which will result in residual toxicity if the product is not purified. Analysis of the mechanism of TPA may provide a basis for safe and efficient increases in lycopene yield.

**FIGURE 5:**
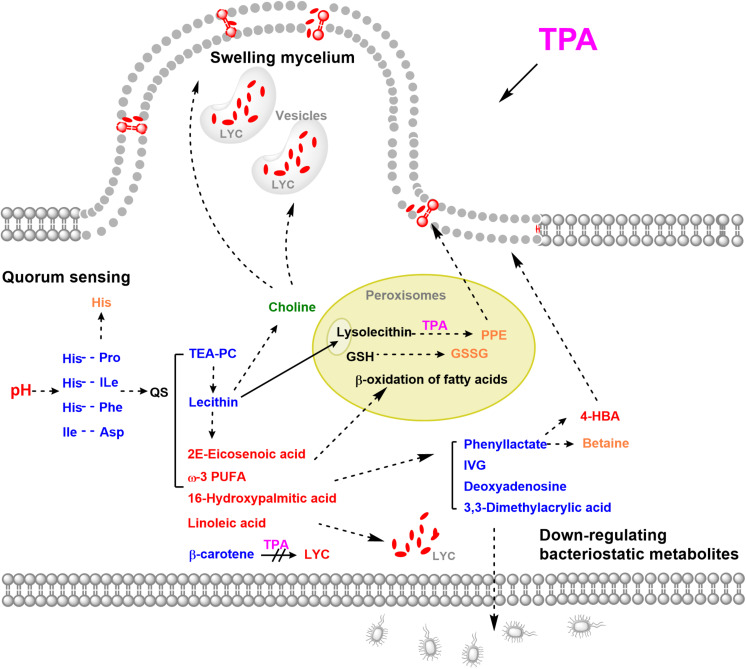
Possible mechanisms of TPA to enhance lycopene in *B. trispora.* Red, up-regulated significantly differential metabolites; Blue, down-regulated significantly differential metabolites; Orange, up-regulated differential metabolites; Green, down-regulated differential metabolites. Abbreviations: 4-HBA, 4-hydroxybenzoate; GSSG, glutathione disulfide; GSH, glutathione; IVG, isovalerylglycine; LYC, lycopene; PPE, 1-palmitoyl-2-hydroxy-sn- glycero-3-phosphoethanolamine; TEA-PC, thioetheramide-PC; TPA, tripropylamine; ω-3 PUFA, 11(Z),14(Z)-eicosadienoic acid.

In this study, to determine the mechanism of TPA-enhanced lycopene accumulation, the untargeted metabolome of *B. trispora* treated with TPA was analyzed. Fifteen significantly differential metabolites were screened, of which 10 were down-regulated and 5 were up-regulated. The down-regulated metabolites were mainly cyclic dipeptides, bacteriostatic compounds, and lipids, while the up-regulated metabolites were mainly unsaturated fatty acids. Through the analysis of differential metabolites, we found that TPA mainly promotes the accumulation of lycopene by initiating quorum sensing (QS), swelling mycelia, and down-regulating bacteriostatic metabolites (Figure 5).

### Quorum Sensing

QS is a complex regulatory network with global control over diverse target functions, including nutrient acquisition, biofilm formation, and motility (Liu et al., 2010; Hallan et al., 2020). Cyclic dipeptides are QS autoinducers and can freely penetrate the cell membrane and activate the QS system (which includes regulation of cell membranes, cell growth, secondary metabolite production, and anti-oxidation) between organisms of the same species (Holden et al., 1999). In specific environments, the higher the number of microorganisms, the higher the concentration of cyclic dipeptide secreted (Wang et al., 2010). However, the stability of cyclic dipeptides is greatly reduced under high pH conditions (Gao et al., 2006). In this study, the extracellular and intracellular pH were both high after treatment with TPA (Figure 3), indicating that the down-regulation of cyclic dipeptides was caused by the increase in pH. Additionally, the up-regulation of His further increased intracellular pH. Thus, we speculated that TPA could enhance lycopene accumulation not only by the inhibition of the β-carotene cyclization, but also by the down-regulation of cyclic dipeptides, which induced the increase of intracellular and extracellular pH, and thereby starting QS.

### Swelling Mycelia

The cell membrane is mainly composed of phospholipids and proteins. Approximately, 70–80% of the phospholipids are phosphatidylethanolamine, and an increase in its content will increase the volume of the cell membrane. In this study, TEA-PC and lecithin were down-regulated and unsaturated fatty acids were up-regulated after treatment with TPA. This effect may have been caused by the hydrolysis of lecithin by phospholipase. TEA-PC is an inhibitor of the competitive reversible secretory phospholipase A2, which can catalyze the hydrolysis of ester bonds at the sn-2 position of lecithin, producing free fatty acids and lysolecithin (Kita et al., 2019). Unsaturated fatty acids have antioxidant effects, and the solubility of lycopene in unsaturated fatty acids is high (Failla et al., 2014). Furthermore, linoleic acid can enhance the production of red pigments in *Monascus ruber* by accelerating the cAMP-PkA pathway (Huang et al., 2017). This indicates that TPA can increase the solubility of lycopene in cells and promote lycopene production through the induction of linoleic acid. However, in this study, lysolecithin did not show differential levels, and its derivative PPE had the greatest up-regulation in differential metabolites.

Furthermore, tertiary amines will first act on the lipid region of the cell membrane (Zhang et al., 2007; Hermetter et al., 2013). Thus, we speculated that the up-regulation of PPE was caused by the direct action of TPA on lecithin. Moreover, PPE can shuttle through the cell membrane, relax the local phospholipid bilayer, and accumulate β-carotene in the cell membrane, thereby increasing its production (Wu et al., 2017). These effects indicate that, in this study, up-regulated PPE could increase the amount of the cell membranes and penetrate the phospholipid bilayer to relax the cell membrane.

Among the differential metabolites, the down-regulation of choline and up-regulation of downstream betaine suggest that choline levels increase to produce betaine in glycine, serine, and threonine metabolism (Supplementary Figure 3). Betaine is a quaternary amine alkaloid, which can donate methyl with high activity, promote lipid metabolism, and thereby promote cell growth. Furthermore, choline is a component of cell membrane, and changes in its content are closely related to the morphology of filamentous fungi (Markham et al., 1993). When the concentration of choline decreases, the hyphae branch and expand, and many cytoplasmic vesicles accumulate locally (Markham et al., 1993). In this study, choline was down-regulated, the hyphae swelled, and many localized small vesicles were observed (Figure 4). We speculated that this morphological change in hyphae is beneficial for the accumulation of lycopene in the cell membrane and intracellular vesicles.

### Down-Regulating Bacteriostatic Metabolites

Interestingly, among the significantly differential metabolites, bacteriostatic phenyllactic acid, IVG, 3,3-dimethylacrylic acid, and deoxyadenosine were all down-regulated. Phenyllactic acid is a recently discovered type of natural bacteriostatic metabolite that can inhibit the growth of bacteria such as *E. coli* and *Listeria monocytogenes* (Feasley et al., 2006). It is also a substrate for the synthesis of betaine and 4-HBA, which were up-regulated in this study. Moreover, both betaine and 4-HBA affect fungal morphogenesis and promote mycelial growth (Chong et al., 2009; Zou et al., 2016). Our findings indicate that the metabolic flow to lipid metabolism and mycelial growth were increased, and the bacteriostatic metabolic flow was reduced.

The differences in *E. coli* densities between the TPA and control groups, shown in Figure 3A, may be because TPA itself has certain bacteriostatic abilities and it could transport into cell for binding cyclase (Wang et al., 2016a) and lecithin (Hermetter et al., 2013). The detected data on bacteriostatic ability showed that the antibacterial ability of TPA-treated *B. trispora* was indeed weakened. This may be a manifestation of *B. trispora* maintaining its own metabolic balance, which also reminds us to pay attention to the aseptic operation when using TPA to increase lycopene production.

Furthermore, most unsaturated fatty acids were up-regulated, and the differential metabolite GSSG was up-regulated 1.24-fold (Supplementary Table 2). GSSG is produced by the oxidation of GSH in peroxisomes, where the β-oxidation of fatty acids is also carried out, suggesting that peroxisomes play an important role in the action of TPA. Furthermore, peroxisomes can relieve the toxicity of amines through transamination (Nazarko et al., 2007), which suggests that the up-regulated PPE in this study may be produced by transamination between TPA and lysolecithin in peroxisomes.

The correlation analysis of differential metabolites showed that cyclic dipeptides have significant positive correlations with cell membrane-related TEA-PC and lecithin and bacteriostatic ability related to 3,3-Dimethylacrylic acid and deoxyadenosine; they also have a significant negative correlation with peroxisome-related GSSG, cell membrane-related PPE, and choline (Figure 2). Our data suggest that TPA can enhance lycopene accumulation not only by inhibiting the cyclization of β-carotene but also by down-regulating cyclic dipeptides to initiate QS. Other effects of TPA include the up-regulation of unsaturated fatty acids, PPE, and 4-HBA and down-regulation of choline and bacteriostatic metabolites for metabolic flux redistribution, locally swelling mycelia with vacuoles, and high lycopene capacity.

## Data Availability Statement

The original contributions presented in the study are included in the article/Supplementary Material, further inquiries can be directed to the corresponding author/s.

## Author Contributions

YaW analyzed the data in the “Discussion” section and drafted the manuscript. YuW analyzed the data in the “Results” section and provided experimental design guidance. YiW and XC did the fermentation experiments. CL and KL did the biochemical detection experiments. MZ did the experimental design guidance. YZ and ZL modified the manuscript. All authors contributed to the article and approved the submitted version.

## Conflict of Interest

The authors declare that the research was conducted in the absence of any commercial or financial relationships that could be construed as a potential conflict of interest.

## Footnotes

1 https://www.genome.jp/kegg/pathway.html

## Supplementary Material

Supplementary Figure 1 The total ion chromatograms of CG2 and TPA3 in positive and negative mode. Pos, positive mode; Neg, negative mode; TPA, tripropylamine treated group; CG, control group.

Supplementary Figure 2 The score chart of PCA and PLS-DA analyses. Pos, positive mode; Neg, negative mode; PC 1, principal component 1; PC 2, principal component 2; TPA, tripropylamine treated group; CG, control group.

Supplementary Figure 3 Glycine, serine and threonine metabolism in TPA treated *B. trispora.* Orange, up-regulated differential metabolites; Green, down-regulated differential metabolites.

Supplementary Table 1 Differential metabolites between TPA and CG in positive and negative modes.

Supplementary Table 2 Described differential metabolites between TPA and CG in positive and negative modes.
