# Supplementary figures and images for: Untargeted Global Metabolomic Analysis Reveals the Mechanism of Tripropylamine-Enhanced Lycopene Accumulation in Blakeslea trispora
Source: Front Bioeng Biotechnol. 2021 Jun 2;9:673225. doi: 10.3389/fbioe.2021.673225 (PMC8207141; doi:10.3389/fbioe.2021.673225)

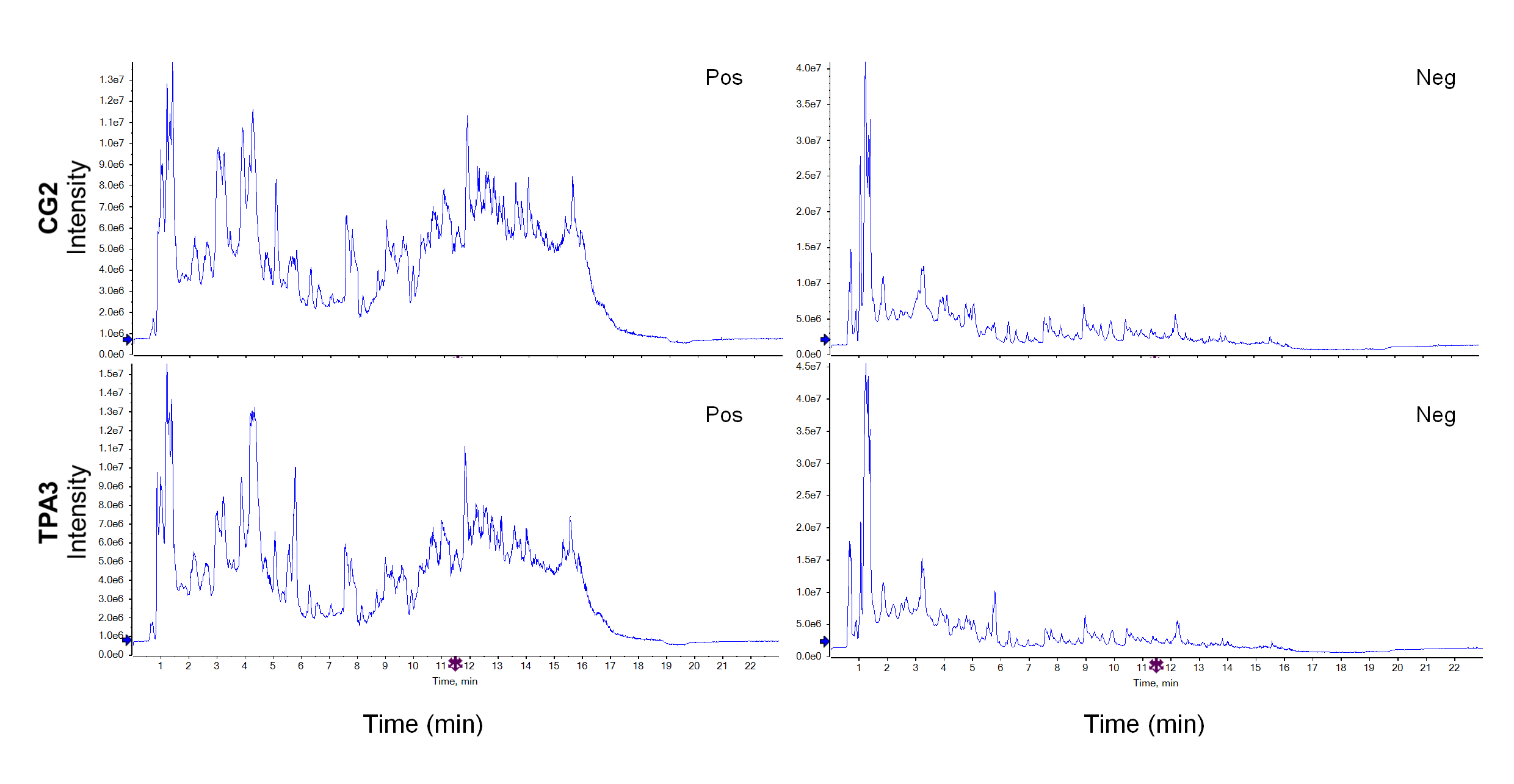

Supplement: Supplementary Figure 1 — The total ion chromatograms of CG2 and TPA3 in positive and negative mode. Pos, positive mode; Neg, negative mode; TPA, tripropylamine treated group; CG, control group. [file Image_1.TIF]

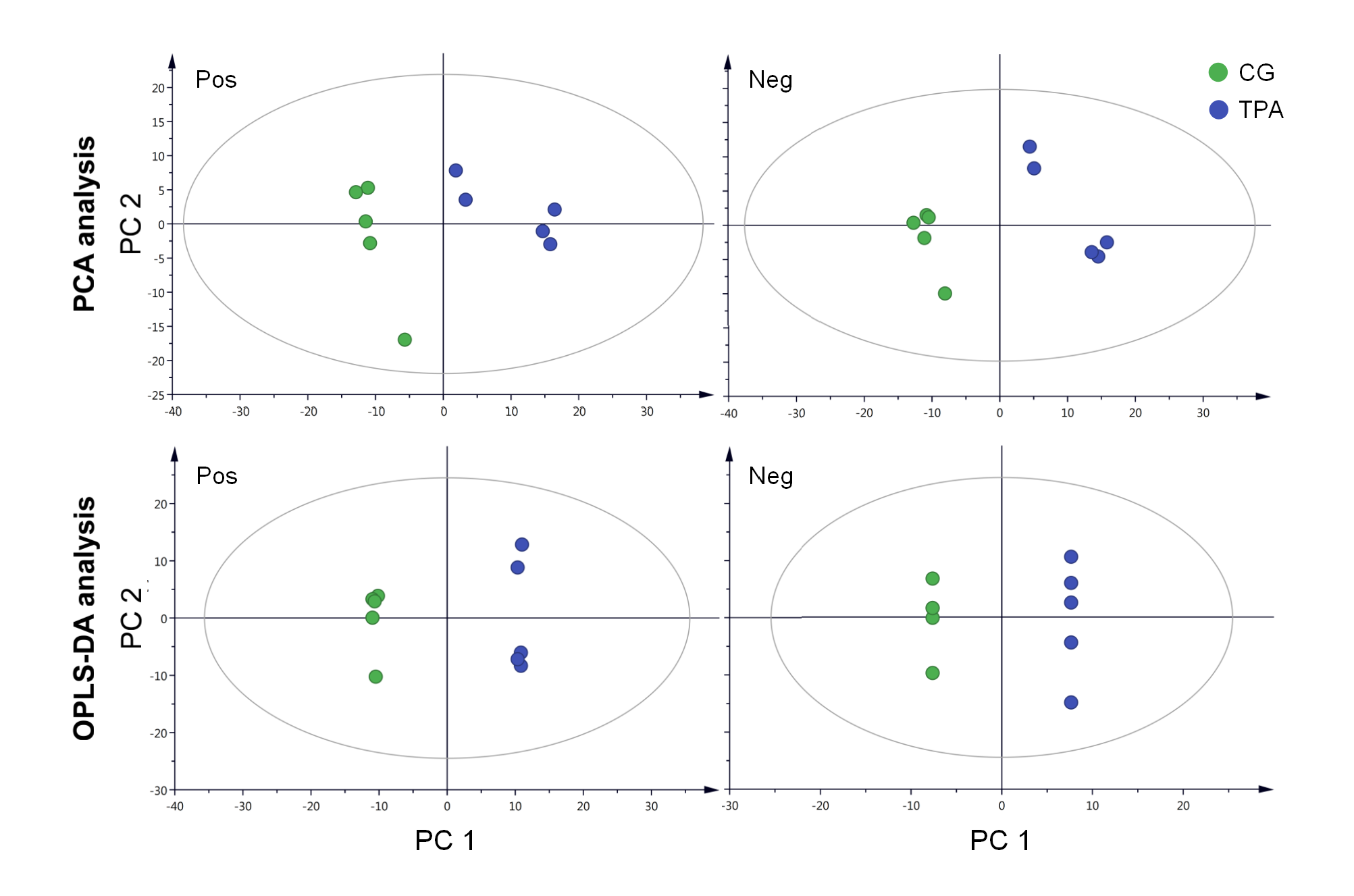

Supplement: Supplementary Figure 2 — The score chart of PCA and PLS-DA analyses. Pos, positive mode; Neg, negative mode; PC 1, principal component 1; PC 2, principal component 2; TPA, tripropylamine treated group; CG, control group. [file Image_2.TIF]

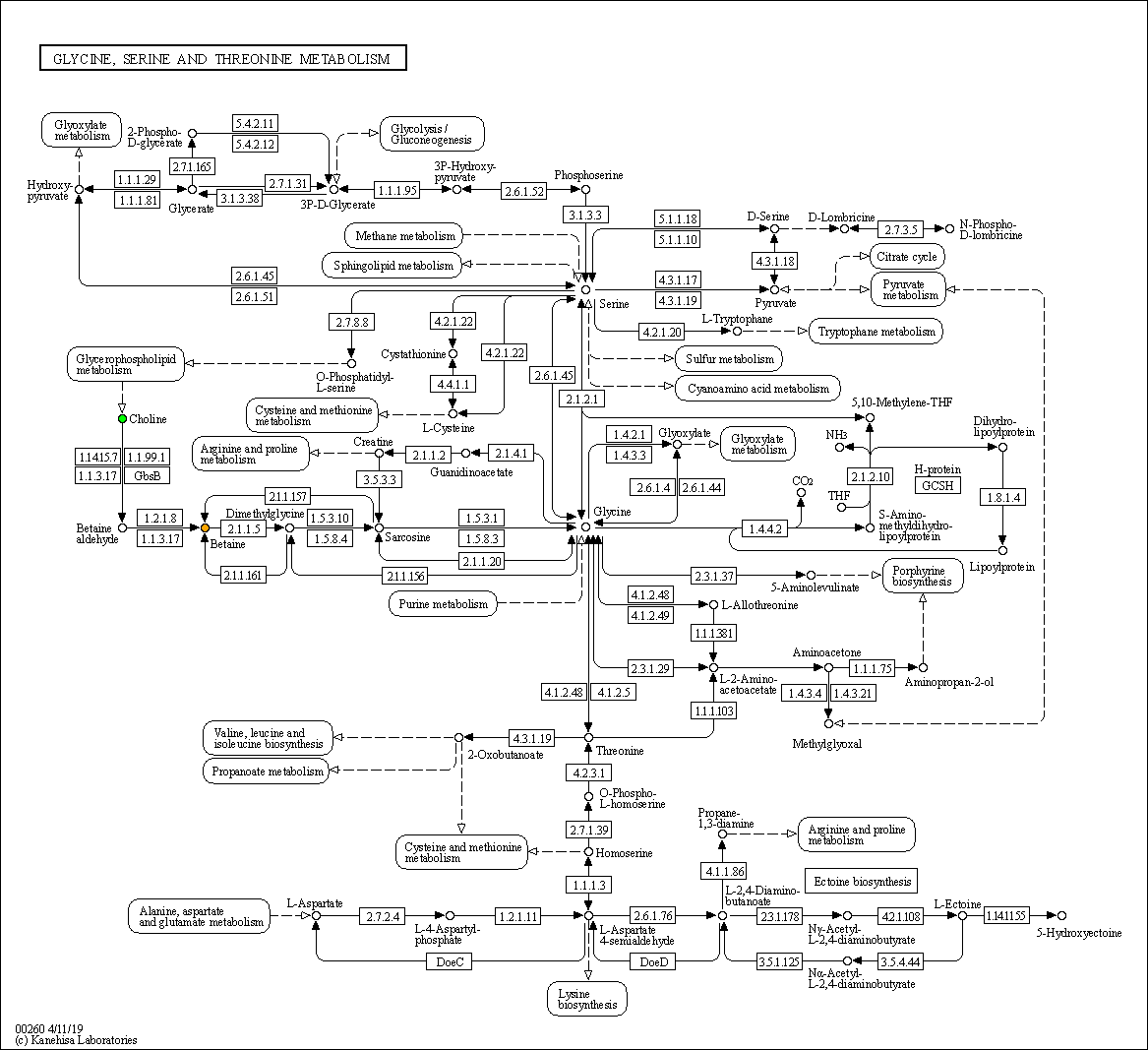

Supplement: Supplementary Figure 3 — Glycine, serine and threonine metabolism in TPA treated B. trispora. Orange, up-regulated differential metabolites; Green, down-regulated differential metabolites. [file Image_3.PNG]
